# Proposal of a Nutritional Quality Index (NQI) to Evaluate the Nutritional Supplementation of Sportspeople

**DOI:** 10.1371/journal.pone.0125630

**Published:** 2015-05-04

**Authors:** Alba Palacin-Arce, Celia Monteagudo, Juan de Dios Beas-Jimenez, Fatima Olea-Serrano, Miguel Mariscal-Arcas

**Affiliations:** 1 Research Group on Nutrition, Diet and Risk Assessment-AGR255, Nutrition and Food Science Department, University of Granada, Granada, Spain; 2 Andalusian Centre of Sport Medicine (CAMD), Junta de Andalucía, Spain; 3 Food Technology, Nutrition and Food Science Department, University of Murcia, Murcia, Spain; National Institute of Agronomic Research, FRANCE

## Abstract

**Background:**

Numerous supplements are used by sportspeople. They are not always appropriate for the individual or the sports activity and may do more harm than good. Vitamin and mineral supplements are unnecessary if the energy intake is sufficient to maintain body weight and derives from a diet with an adequate variety of foods. The study objectives were to evaluate the main nutrients used as supplements in sports and to propose a nutritional quality index (NQI) that enables sportspeople to optimize their use of supplements and detect and remedy possible nutritional deficits.

**Material and Methods:**

A nutritional study was performed in 485 sportspeople recruited from *Centros Andaluces de Medicina del Deporte*, (CAMD). All completed socio-demographic, food frequency, and lifestyle questionnaires. The nutritional quality of their diet and need for supplementation were evaluated by scoring their dietary intake with and without supplementation, yielding two NQI scores (scales of 0-21 points) for each participant.

**Results:**

A superior mean NQI score was obtained when the supplements taken by participants were not included (16. 28 (SD of 3.52)) than when they were included (15.47 (SD: 3.08)), attributable to an excessive intake of some nutrients through supplementation.

**Conclusions:**

These results indicate that sportspeople with a varied and balanced diet do not need supplements, which appear to offer no performance benefits and may pose a health risk.

## INTRODUCTION

The potential health hazards of sedentary life and the health benefits of increased daily physical exercise have long been recognized. The very high and growing prevalence of obesity has prompted international bodies (WHO, FAO, etc.) to launch multiple initiatives to encourage a healthy diet and daily physical activity[1,2]. Millions of individuals worldwide now practice regular exercise to prevent or combat disease and enhance their quality of life or to improve their physical condition for participation in sports activities[3].

The performance of sportspeople is influenced by numerous physical, psychological, nutritional, and environmental factors. Nutritional status plays a key role, and minor dietary errors at critical times can ruin months or even years of hard training[4]. In fact, as the margin between victory and defeat becomes ever narrower, sportspeople increasingly resort to nutritional supplements[5,6]. There has been an exponential growth over recent years in the consumption of supplements, largely composed of proteins, amino acids, vitamins and minerals, and this has been especially marked among sportspeople[7].

Many supplements are promoted, with no sound scientific foundation, for enhancing performance and muscle mass as part of normal training or competition routines and for facilitating recovery after training or sports injury[8]. Around 85% of elite athletes are reported to take supplements, including vitamins, minerals, proteins, and/or creatinine, among others. These should be used with care in order to minimize risks, but it appears that sportspeople rarely wait for convincing proof of their effectiveness or safety[9]. In general, vitamin and mineral supplements are not considered necessary if the usual diet contains an appropriate variety of foods that provide sufficient energy to maintain the body weight[10,11]. However, supplements may be required by sportspeople who restrict their energy consumption or remove one or more food groups in order to lose weight or by those whose diets are unbalanced and deliver a low density of micronutrients[12].

Sportspeople frequently consume supplements that are not always appropriate for their bodies or for their sports activity and that may have no effect or may even exert adverse effects[9]. The American College of Sports Medicine, American Dietetic Association, and Dieticians of Canada have issued warnings about the frequently incorrect and unsafe utilization of nutritional ergogenic aids, which should only be taken after careful evaluation of their safety, effectiveness, potency, and legality; it was emphasized that individual nutritional guidance and advice should be based on a complete nutritional assessment by experts[12]. Nutritional indexes used for this purpose should evidently be as comprehensive as possible, and there is a need for an instrument that takes account of the consumption of supplements and includes all micronutrients (e.g., ramified or sulphured amino acids).

The objectives of this study were to evaluate the main nutrients used as supplements in sports and to propose a nutritional quality index (NQI) that enables sportspeople and other groups to optimize their use of supplements and detect and remedy possible nutritional deficits. Two NQI scores were obtained for each individual: one based on FFQ data alone and the other also including nutrient intake from supplements, as recorded in the life habits questionnaire, with the aim of comparing and evaluating the advantages or drawbacks of the supplement intake of the participants.

## MATERIALS AND METHODS

### Participants

Volunteers were recruited from among affiliated members of the eight Andalusian Sports Medicine Centers (CAMD, *Centros Andaluces de Medicina del Deporte*) and of the Sierra Nevada High Altitude Training Centre (CAR, Centro de Alto Rendimiento) in Southern Spain. Each participating centre invited its members to participate in the study, with the incentive that volunteers would each receive a report on their nutritional status and diet upon its completion. The study inclusion criteria were a good health status, the practice of sports on at least two days/week, and informed consent to participation in the study, which was conducted in accordance with the Declaration of Helsinki (October 2000) and approved by the ethics committee of the University of Granada. Alphanumeric codes were assigned to participants to preserve their anonymity in the data treatment.

### Questionnaire

Participants were administered with a document containing: a questionnaire on their socio-demographic characteristics (including sports activities), a semi-quantitative food frequency questionnaire (FFQ), and a questionnaire on life habits (e.g., smoking, eating/drinking, consumption of products to improve performance). The socio-demographic data gathered were: province, sex, age, marital status, educational level, profession, place of work and sports activity, including the modality, level and phase of the season (e.g., competition, training, etc.).

The FFQ has been validated and used in numerous studies[13–15]. It records the consumption or not of each food, the number of times consumed per day, week, or month during the previous year, and the amount consumed each time in g, mL, or domestic measures (e.g., platefuls, glassfuls, tea/table spoonfuls, etc). The daily food and nutrient intake was calculated (in g or mL) from the FFQ by multiplying the standard serving size of each item by the value corresponding to the consumption frequency: never = 0; 1–3 times/ month = 0.07; 1–2 times/week = 0.21; 3–4 times/ week = 0.50; 5–6 times/ week = 0.80; 1 time/day = 1; and 2–3 times/day = 2.5 [16–18]. These data were processed by using the Dial version 1.19 diet program[19]. Results were analysed by food group, considering 12 main groups, i.e., cereals, dairy products, eggs, meat, fish, legumes, vegetables, fruits, sweets, fats/oils, alcohol and stimulants.

### Proposed NQI for supplementation (NQIs)

The index is based on the daily recommended intake[20] and daily tolerable upper intake level (UL) for the product/supplement in question, as recommended by the WHO and numerous authors[21–27]. In the case of protein, there is no established UL, which was considered to be two-fold the recommended intake for sportspeople[28] in the present study. The optimal intake of each nutrient was considered to be between 2/3 of the recommended intake and the UL value; intake within this range was scored as 1 and intake below or above this range was scored as 0. The score ranged between 0 and 21 points. The nutrients considered were: total proteins and amino acids (valine, leucine, isoleucine, and sulphured amino acids), B group vitamins (B_1_, B_2_, B_3_, B_6_, folic acid), vitamin A, vitamin D, vitamin C, and vitamin E) and mineral salts (calcium, iron, selenium, zinc, copper, and iodine and magnesium). Two scores were obtained for each study participant: one derived from the sum of the values obtained from the FFQ for the different nutrients consumed in the daily diet; and the other also including the values of the nutrients consumed in supplements, as recorded in the life-habits questionnaire.

### Statistical analysis

SPSS-19 (IBM Inc. Chicago, IL, USA) was used for the data analyses, applying the Student’s t-test and ANOVA for the comparison of means and performing stepwise linear regression. The statistical tests applied are given in the table footnotes. P< 0.05 was considered significant in all tests.

## RESULTS

Study inclusion criteria were met by a total of 515 volunteers. After exclusion of those who failed to complete the questionnaires in a correct manner, the final study sample comprised 485 healthy sportspeople (72.2% males and 27.4% females) aged between 18 and 69 yrs. The mean age was 27.95 (SD 11.69) yrs in the males and 19.89 (SD 7.83) yrs in the females.

Table 1 lists the estimated value reported for each nutrient considered in the index (without and with supplementation) alongside the percentage of the dietary reference intake (DRI) to which it corresponds (DRI source is specified in the footnotes). The mean intake of all nutrients was >100% of the DRI, with the exception of valine, leucine, methionine/cysteine and vitamin D. The mean intake of all nutrients significantly differed from the mean DRI, with the exception of iodine, selenium and methionine/cysteine (p>0.05). The mean value of the index was 16.28 when supplements were not included and 15.49 when they were. For both indexes, the lowest value was 2 points and the highest was 21. In the males, the mean score was 16.47 (SD 3.14) without *versus* 15.69 (SD 2.85) with supplement intake, and in the females, it was 16.09 (SD 3.31) without *versus* 15.19 (SD 3.15) with supplement intake, with no significant gender differences.

**Table 1:** Intake of nutrients with respect to Dietary Reference Intake (DRI) and NQI mean value for both groups.*

Nutrient	Dietary intake		Dietary intake + Supplements		
	Mean (SD)	% DRI	Mean (SD)	% DRI	*p* †
Protein (g/d)	141.03 (56.90)	137.3	143.02 (57.59)	160.0	<0.001
Valine (mg/g protein)	29.68 (13.43)	92.7	31.33 (18.06)	97.9	<0.05
Isoleucine (mg/g protein)	27.36 (12.49)	109.4	29.10 (18.47)	116.4	<0.05
Leucine (mg/g protein)	43.49 (19.94)	85.3	44.22 (19.79)	86.7	<0.05
Methionine&Cysteine (mg/g protein)	23.87 (34.64)	82.9	21.68 (13.00)	83.2	0.318
Calcium (mg/d)	1486.35 (559.59)	148.6	1491.45 (561.18)	149.1	<0.05
Iron (mg/d)	27.11 (12.60)	288.9	27.26 (12.64)	290.3	<0.001
Iodine (µg/d)	178.14 (70.49)	118.8	179.72 (74.58)	119.8	0.188
Magnesium (mg/d)	585.07 (219.09)	153.91	592.18 (221.80)	155.7	<0.001
Selenium (µg/d)	177.81 (82.33)	323.3	201.28 (486.51)	366.0	0.303
Zinc (mg/d)	18.04 (7.01)	180.1	18.25 (7.07)	182.1	<0.001
Copper (mg/d)	1.40 (0.45)	155.8	1.40 (0.44)	155.8	<0.001
Vitamin B_1_ (mg/d)	2.62 (1.07)	223.7	2.71 (1.16)	231.1	<0.001
Vitamin B_2_ (mg/d)	3.30 (1.34)	265.7	3.38 (1.38)	271.7	<0.001
Vitamin B_3_ (mg/d)	39.13 (18.85)	253.8	39.91 (19.69)	258.84	<0.05
Vitamin B_6_ (mg/d)	4.51 (1.81)	346.9	4.66 (1.92)	358.3	<0.001
Folic acid (µg/d)	623.82 (276.45)	156.0	627.50 (280.10)	156.9	<0.05
Vitamin C (mg/d)	319.46 (159.16)	373.0	323.55 (162.18)	377.6	<0.001
Vitamin A (µg/d)	960.36 (537.90)	114.9	989.01 (555.61)	118.4	<0.001
Vitamin D (µg/d)	7.04 (4.78)	47.0	7.24 (4.95)	48.3	<0.001
Vitamin E (mg/d)	17.04 (8.33)	113.6	17.60 (8.74)	117.3	<0.001
NQI**	**16.28 (3.22)**	**-**	**15.48 (3.00)**	**-**	**<0.001**

* Dietary Reference Intakes for Energy, Carbohydrate, Fibre, Fat, Fatty Acids, Cholesterol, Protein and Amino Acids (2002/2005). Washington DC: National Academy Press.

** NQI: Nutritional Quality Index

† Student’s t-test for paired samples.

Table 2 shows the consumption of each nutrient in tertiles: first, above the UL; second, between 2/3 of the recommendation and the UL, and third below 2/3 of the recommendation. The consumption of each intake was in the third tertile for >60% of participants.

**Table 2:** Distribution of the population (%) according to the relationship of nutrient intake with UL and DRI values.

		≥ UL	2/3 DRI-UL	< 2/3 DRI
Protein	DI	-	96.5	3.5
	DI + S	59.5	39.0	1.5
Valine	DI	5.4	76.1	18.5
	DI + S	6.4	76.9	16.7
Isoleucine	DI	1.5	63.4	35.1
	DI + S	1.5	65.1	33.4
Leucine	DI	1.5	63.4	35.1
	DI + S	1.5	65.1	33.4
Methionine &Cysteine	DI	3.8	58.7	37.5
	DI + S	2.9	60.3	36.8
Calcium	DI	4.8	89.8	5.4
	DI + S	4.8	90.0	5.2
Iron	DI	8.7	88.5	2.8
	DI + S	8.7	88.8	2.5
Iodine	DI	-	91.3	8.7
	DI + S	-	91.3	8.7
Magnesium	DI	90.4	5.3	4.4
	DI + S	90.6	5.3	4.1
Selenium	DI	2.3	95.5	2.3
	DI + S	2.5	95.2	2.3
Zinc	DI	0.9	96.3	2.8
	DI + S	0.9	96.3	2.8
Copper	DI	-	99.0	1.0
	DI + S	-	99.0	1.0
Vitamin B_1_	DI	53.3	44.1	2.5
	DI + S	55.5	41.9	2.6
Vitamin B_2_	DI	67.0	31.7	1.4
	DI + S	68.7	29.9	1.4
Vitamin B_3_	DI	68.6	29.1	2.3
	DI + S	69.1	28.7	2.3
Vitamin B_6_	DI	-	97.6	2.4
	DI + S	-	94.2	5.8
Folic acid	DI	10.4	83.7	5.9
	DI + S	11.1	83.0	5.9
Vitamin C	DI	-	96.6	3.4
	DI + S	-	96.6	3.4
Vitamin A	DI	-	75.2	24.8
	DI + S	-	76.6	23.4
Vitamin D	DI	-	19.6	80.4
	DI + S	-	21.0	79.0
Vitamin E	DI	-	86.4	13.6
	DI + S	-	82.2	17.8

DI = Dietary Intake; DI + S = Dietary Intake + Supplements.

Stepwise regression analysis revealed that only nine of the items significantly contributed to the indexes; these were the same items for both indexes but made distinct percentage contributions to the scores. For instance, protein accounted for 35% of the index score when supplements were not included compared with only 6% when they were (Table 3).

**Table 3:** Nutrients that together contribute to > 95% of the NQI scores (stepwise regression).

Dietary intake*		Dietary intake +Supplements	
Nutrients	R^2^	Nutrients	R^2^
Protein	0.355	Zinc	0.381
Isoleucine	0.560	Leucine	0.616
Zinc	0.679	Folic acid	0.712
Vitamin A	0.748	Vitamin A	0.777
Leucine	0.805	Protein	0.832
Folic acid	0.861	Isoleucine	0.867
Vitamin D	0.901	Iron	0.903
Iron	0.939	Vitamin D	0.938
Niacin	0.957	Niacin	0.955

Logistic regression analysis (Table 4) showed that the use of nutritional supplementation was influenced by educational level, type of sport (group v. individual), current or past smoking habit and the history of a dietary regime. There was a greater likelihood of supplementation in participants with a medium educational level (OR = 0.36, 95%CI: 0.13–1.04), those involved in group sports (OR = 2.59, CI 95%: 0.95–7.03), non-smokers (OR = 4.20, 95%CI: 0.91–19.48) and those who had never been on a diet (OR = 2.52, 95%CI: 0.94–6.78).

**Table 4:** Factors influencing the intake of supplements in the study population (logistic regression).

		OR	CI 95%		p
Fat (%)		0.964	0.90	1.04	0.322
Education level*	Low (Ref.)				0.165
	Medium	0.36	0.13	1.04	0.048
	High	0.62	0.25	1.58	0.317
Group sport	No(Ref.)				
	Yes	2.59	0.95	7.03	0.052
Period of season	Pre-season (reference)				0.397
	Competition	1.18	0.36	3.87	0.781
	Recovery/Transition	1.88	0.68	5.16	0.222
Smoker	Yes (Ref.)				
	No	4.20	0.91	19.48	0.047
Ex-smoker	Yes (Ref.)	0.37	0.13	1.04	0.050
	No				
Dietary regime	Often (Ref.)				0.221
	Sometimes	1.43	0.26	7.86	0.680
	Once	0.41	0.04	4.42	0.460
	Never	2.52	0.94	6.78	0.048
“Light” products	Yes (Ref.)				0.739
	No	1.26	0.50	3.21	0.627
	Sometimes	0.87	0.35	2.12	0.754
Comprehension of nutritionalinformation on the label	Yes (Ref.)				0.632
	No	0.75	0.32	1.75	0.501
	Sometimes	0.55	0.15	2.07	0.378

Reference: yes to supplementation

* low = primary schooling; medium = secondary schooling/vocational diploma, *high = university studies*.

## DISCUSSION

There has been a continuing increase in the use of supplements, especially by sportspeople[5]. In the present study of 485 healthy active sportspeople, around two-thirds consumed some type of supplement, mostly preparations based on proteins, ramified amino-acids, vitamins and/or minerals, similar to reports by other researchers[29].

Macronutrient and micronutrient requirements may be higher in sportspeople, due to their physical exertions, but these were amply met by the daily diet of almost all of the participants. Thus, the requirement for protein was met by the dietary intake of almost all (96.5%) of the participants; however, 59.5% of the participants exceeded the UL for protein by taking supplements, potentially putting their health at risk. A study in the UK associated a protein intake above the UL with various adverse effects, and concluded that it should not exceed two-fold the recommended level[20,22]. Other authors claimed that a protein intake >35% of the energy intake has no adverse effects but that a percentage above 45% can be life-threatening if maintained for several weeks[30]. It should be taken into account that the protein UL values considered in the present study were two-fold higher than the recommendations for adult sportspeople, which are much higher than those for the general population[31].

Because the replacement level of minerals is low, deficits can usually be avoided by consuming a healthy and balanced diet. Nevertheless, athletes frequently have problems in this respect due to increased excretion (from exercise or other factors) and problems of intestinal absorption[32]. This is mainly a concern with iron and calcium, whose deficit can be responsible for anaemia and osteoporosis, among other diseases, although long-term supplementation may do more harm than good[33]. The dietary intake of minerals was adequate for the majority of participants in the present study, and the use of supplements offered no advantage. In fact, supplementation increased the percentage of participants with an excessive intake of magnesium (>UL).

Vitamins cannot be synthesized and must therefore be obtained daily in the diet. Sportspeople require more vitamins than sedentary people due to their higher energetic metabolism. However, they generally consume more food to compensate for the greater caloric output generated by exercise, thereby increasing their vitamin intake. Consequently, vitamin deficits are rare unless the diet quality is inadequate, and supplements are unnecessary. Furthermore, there is increasing evidence from animal and human studies that supplementation with antioxidants can have a negative effect on sports performance[34–36]. On the other hand, the dietary vitamin D intake was below 2/3 of the DRI in 80% of our study participants, and an intake between this value and the UL was achieved by some of these through supplementation. Table 1 depicts the percentage of the mean value of all nutrients, showing that all DRIs are exceeded except for vitamin D. The mean intakes of selenium, vitamin C, vitamin B_2_, niacin (B_3_), and B_6_ were between 2.5- and 3.8-fold higher than recommended levels. Recent studies have reported that nutritional supplementation does not offer a clear benefit for patients with chronic diseases and may even have negative effects; nevertheless. It was recently concluded in widely-read editorial in the Annals of Internal Medicine[37] that although available evidence does not rule out minor or major benefits or harm in small population subgroups, it is well established that the addition of (most) mineral or vitamin supplements to the diet of well-nourished adults offers no benefits and might even be harmful.

Study limitations include the preponderance of male participants, although this reflects the fact that the regular performance of intense physical activity is more than two-fold more frequent among males than among females, at least in Spain[38]. There is also a need for further research on specific sports disciplines and types of participant (e.g., professional or amateur).

A major strength of our index of nutrient intake is that it can be applied to any population group, not solely sportspeople. Supplements are marketed to the general population and are used in work and study settings and for “self-medicating” purposes. The benefits of these supplements are poorly understood and may be outweighed by their negative effects. Consumers may often be unaware of their components or their action mechanism. The method proposed here allows evaluation of the need for supplementation and the potential adverse effects associated with specific nutrients in any individual.

In conclusion, supplementation appears to offer no performance benefits to healthy active sportspeople and may pose a health risk. We propose a method to estimate the quality of diets and the need for supplementation by using the same index to rate the intake of individuals with and without supplementation. Our findings confirm that sportspeople do not need nutritional supplementation if they follow a varied and balanced diet.
